# Development of a Unified Reversed-Phase HPLC Method for Efficient Determination of EP and USP Process-Related Impurities in Celecoxib Using Analytical Quality by Design Principles

**DOI:** 10.3390/molecules25040809

**Published:** 2020-02-13

**Authors:** Tim Tome, Zdenko Časar, Aleš Obreza

**Affiliations:** 1 Faculty of Pharmacy, University of Ljubljana, Aškerčeva c. 7, SI-1000 Ljubljana, Slovenia; tim.tome@sandoz.com; 2 Analytics Department, Sandoz Development Center Slovenia, Lek Pharmaceuticals d.d., Verovškova ulica 57, SI-1526 Ljubljana, Slovenia

**Keywords:** RP-HPLC, AQbD, HPLC method development, HPLC method optimization, pharmacopeia, celecoxib

## Abstract

This article presents the development of a reversed-phase (RP) high-performance liquid chromatographic (HPLC) method for determination of process-related impurities in a celecoxib drug substance following Analytical Quality by Design (AQbD) principles. The method from European Pharmacopeia (EP) for celecoxib drug substance does not sufficiently separate celecoxib from its EP impurity B because the system suitability criterion is not achieved (resolution NLT 1.8). The same issue was observed with the proposed method from United States Pharmacopeia (USP) for celecoxib capsules, where EP impurity A elutes under the main peak. A new HPLC method was developed that eliminates the disadvantages of the two pharmacopeial methods and is capable of efficiently separating and determining all seven impurities listed in EP and the proposed USP monographs. The development of a new HPLC method started with method scouting, in which various C18 and phenyl stationary phases were tested. Improved selectivity was obtained only with a chiral stationary phase. An immobilized Chiralpak IA-3 column used in RP mode turned out to be the most appropriate for method optimization. The ratio of acetonitrile in the mobile phase, flow rate, and column temperature were recognized as critical method parameters (CMPs) and were further investigated using a central composite face response-surface design. A multiple linear regression (MLR) method was applied to fit the mathematical models on the experimental data to determine factor–response relationships. The models created show adequate fit and good prediction abilities. The Monte Carlo simulation method was used to establish the design space. The method developed was verified in terms of precision, sensitivity, accuracy, and linearity, and the results showed that the new method is suitable for determination of seven process-related impurities of celecoxib.

## 1. Introduction

Celecoxib is a selective inhibitor of cyclooxygenase-2 (COX-2) used for the treatment of inflammation, osteoarthritis, rheumatoid arthritis, and pain. It is a member of a non-steroidal anti-inflammatory drug (NSAID) class that has significantly less severe side effects in the gastrointestinal tract, such as gastric ulceration, compared to conventional non-selective NSAIDs. Although long-term treatment may increase the risk of adverse side effects in the cardiovascular system, celecoxib has shown therapeutic effects on colon polyp formation, thus decreasing the risk of colorectal adenoma in patients suffering from familial adenomatous polyposis, which may lead to colorectal cancer [1,2,3,4,5,6].

Several high-performance liquid chromatographic (HPLC) analytical methods for quantitative determination of celecoxib and/or its process-related impurities in drug substance and in formulation (capsule dosage form, microemulsion, niosomal formulation, etc.,) have been reported in the literature [7,8,9,10,11,12,13,14,15,16,17,18,19]. In addition, several methods are known for determination of celecoxib and its metabolites in plasma [20,21,22].

**Figure 1:**
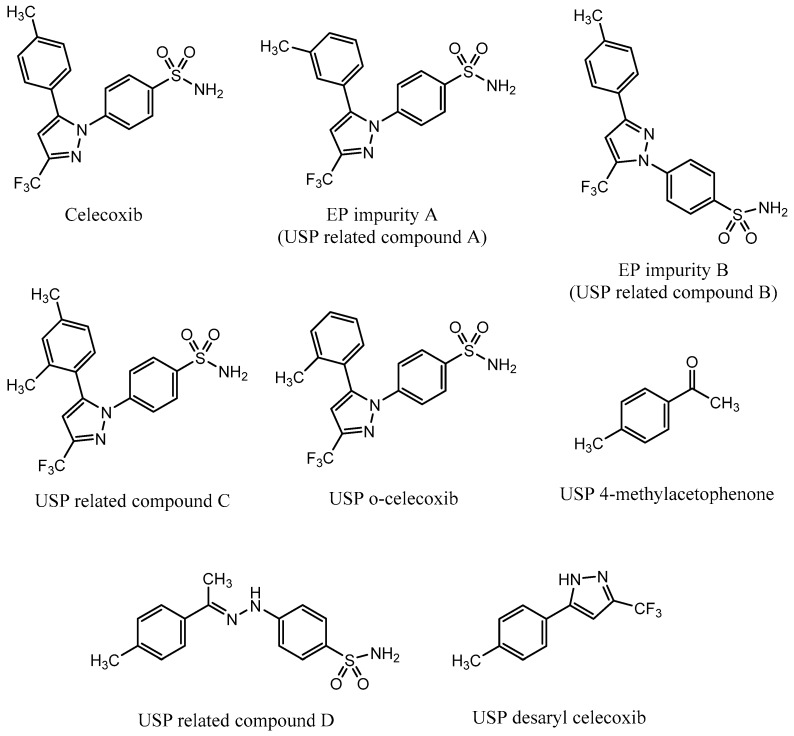
Structure of celecoxib and its process-related impurities defined in European Pharmacopeia (EP) [23] and United States Pharmacopeia (USP) [24,25].

A monograph for control of celecoxib drug substance is available in European Pharmacopoeia (EP), which prescribes an HPLC method for quantitative determination of celecoxib and its two process-related impurities, A and B [23]. EP impurity A is a meta isomer of celecoxib, and EP impurity B is a positional isomer with the benzene sulfonamide functional group positioned on an adjacent nitrogen (Figure 1). The same method is also prescribed in the United States Pharmacopeia (USP) monograph [24].

In April 2017, Pharmacopeial Forum, a USP publication, proposed a new monograph for celecoxib capsules, which introduced an HPLC method for determination of five celecoxib impurities [25]. The listed impurities were USP related compound B, USP related compound C, USP o-celecoxib, USP desaryl celecoxib, and USP 4-methylacetophenone. In addition, USP related compound D was found available as a USP reference standard. USP related compound B is the same impurity as EP impurity B; USP related compound C is an ortho/para isomer of celecoxib; and USP o-celecoxib is an ortho isomer of celecoxib (Figure 1). According to the proposed monograph, the listed impurities are process-related and are controlled in the drug substance.

Some of the published methods [7,10,14,15,18,19] describe determination of assay of celecoxib. It is not reported that the process-related impurities would be taken into account during the method development in these cases. The selectivity of the method for process-related impurities is therefore unknown. Furthermore, other reported methods [8,9,11,12,13,17] describe determination of some process-related impurities of celecoxib (EP impurity B, USP o-celecoxib, USP 4-methylacetophenone), but not all seven pharmacopeial ones. Moreover, none of them mentions the EP impurity A, which turned out as the most challenging impurity to be separated from celecoxib.

Although the existing key methods published in various publications [16,23,24,25] apparently cover the control of process-related impurities in celecoxib, we found that they face several issues that make them unfit for their intended use. The EP and USP [23,24,25] methods appear to be insufficiently selective, whereas the method used by Rao et al. [16] has a very long run time to separate all seven process-related impurities in celecoxib and suffers in terms of sensitivity for the last eluting peak (details are provided in Section 2.1).

The aim of this article was to develop and optimize a selective and robust analytical method for determining seven process-related impurities of celecoxib (Figure 1) with minimal analysis run time. This would enable to cover testing of all seven process-related impurities of celecoxib with a single HPLC method in an efficient and simple manner, thus avoiding separate testing of different sets of impurities with the EP and USP methods. For this purpose, the principles of Analytical Quality by Design (AQbD) were followed [26]. The AQbD approach contributes to achieving an improved method performance with a defined area of method robustness around its optimal working point.

Recently, many publications have been reported discussing the AQbD approach to the development and optimization of HPLC methods, for the determination of either active pharmaceutical ingredient or impurities [26,27,28,29,30,31,32,33,34,35,36,37,38,39,40,41]. Indeed, AQbD has turned out to be a reliable and effective approach, which is reflected in these methods, because they are more robust, easily validated, and have shorter run times for the separation of the same number of analytes compared to methods developed using the one-factor-at-a-time (OFAT) approach [26]. In fact, a new International Conference on Harmonization (ICH) Q14 guideline covering the topic of AQbD is also expected in 2021 [42].

In addition, the method developed has been verified in terms of precision, sensitivity, accuracy, and linearity, and the response factors for seven process-related impurities have been determined.

## 2. Results and Discussion

### 2.1. Initial Testing of Existing Key HPLC Methods

We started our investigation by testing the EP method [23], the proposed USP method from Pharmacopeial Forum [25], and Rao’s method [16] to verify whether these methods have the ability to separate all seven process-related impurities of celecoxib that are stated in EP and USP according to the criteria set in regulatory guidelines.

**Figure 2:**
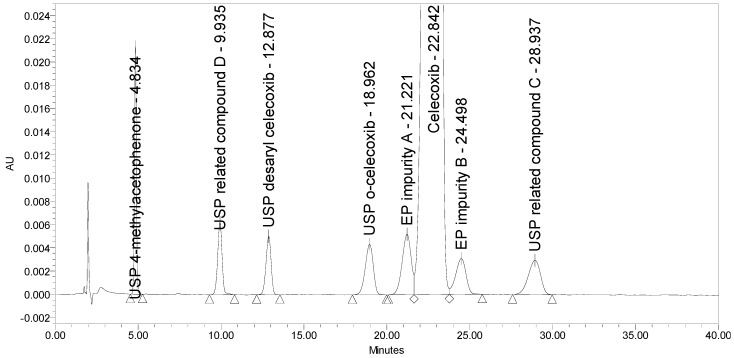
A chromatogram of celecoxib at a concentration of 0.5 mg/mL spiked with EP impurity A, EP impurity B, USP related compound C, USP related compound D, USP o-celecoxib, USP desaryl celecoxib, and USP 4-methylacetophenone at the 0.5% level obtained using the EP method.

The current EP method [23] employs reversed-phase (RP) HPLC separation on a diphenyl stationary phase (Supelcosil LC-DP 5 µm, 250 × 4.6 mm) using an isocratic elution principle with UV detection at 215 nm. The two EP impurities, the four USP impurities, and an additional USP related compound D was injected to test the selectivity of the EP method. Problems with the method were observed. The system suitability requirement for resolution between EP impurity A and celecoxib of NLT 1.5 was barely achieved, and the requirement for resolution between celecoxib and EP impurity B of NLT 1.8 was not achieved. The chromatogram of celecoxib at a concentration of 0.5 mg/mL spiked with seven process-related impurities at the 0.5% level obtained using the EP method is presented in Figure 2.

**Figure 3:**
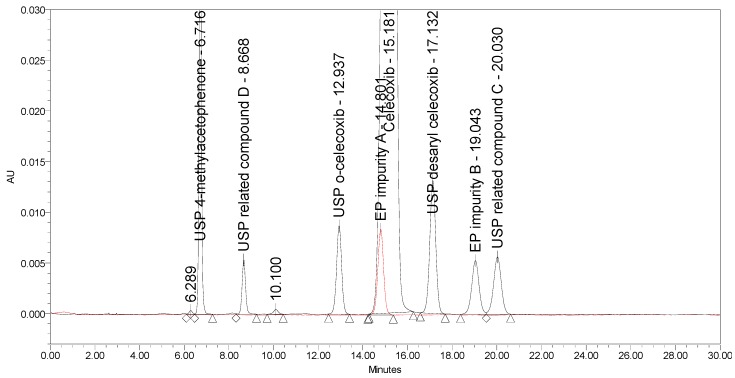
An overlay of chromatograms of celecoxib at a concentration of 1.0 mg/mL spiked with seven investigated impurities at the 0.5% level (black) and of EP impurity A at the 0.5% level (red) obtained using the proposed USP method.

Applying the proposed USP method for celecoxib capsules [25], where the separation occurs on a C18 stationary phase (Peerless Basic C18 5 µm, 250 × 4.6 mm) using an isocratic elution principle with UV detection at 254 nm, a lack of selectivity was observed. EP impurity A eluted under the main peak. Otherwise, the method is capable of separating five listed impurities, as prescribed in the proposed monograph, and an additional USP related compound D, with the prescribed system suitability criteria being confirmed. The chromatograms of celecoxib at a concentration of 1.0 mg/mL spiked with seven process-related impurities at the 0.5% level and of EP impurity A at the 0.5% level obtained using the proposed USP method are presented in Figure 3.

**Figure 4:**
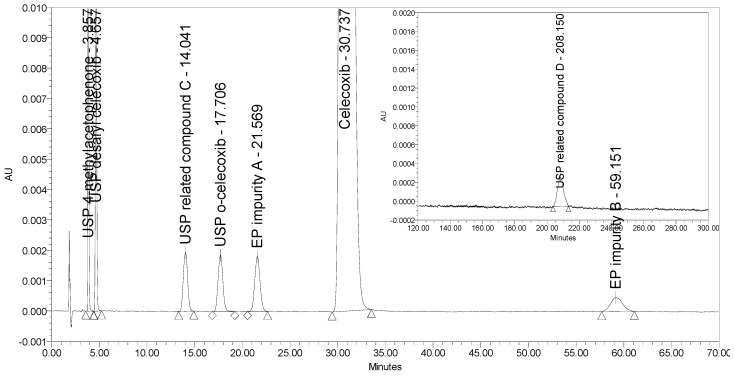
A chromatogram of celecoxib at a concentration of 0.5 mg/mL spiked with six investigated impurities at the 0.5% level (presented from 0 to 70 min) and with USP related compound D at the 2.0% level (presented from 120 to 300 min) obtained using the method by Rao et al.

An HPLC method able to separate and quantify ortho and meta (EP impurity A) isomers of celecoxib has been reported only by Rao et al. [16]. This method employs a normal phase (NP) separation on a chiral stationary phase (Chiralpak AD 10 µm, 250 × 4.6 mm) using an isocratic elution with n-hexane/ethanol mobile phase and UV detection at 255 nm. Although the method turned out to be selective for all seven process-related impurities investigated, the retention time of the last eluting impurity (USP related compound D) was over 200 min. Figure 4 presents the chromatogram of celecoxib at a concentration of 0.5 mg/mL spiked with six process-related impurities at the 0.5% level and USP related compound D at the 2.0% level obtained using the reported method by Rao et al. The selectivity of the method was tested using a Chiralpak AD-3 column with smaller particle size (3 µm) and shorter dimensions (150 × 4.6 mm) as proposed by Rao et al. This means that if using the proposed 250 mm column, the analysis run time would only be prolonged. Moreover, the sensitivity of the method for detection of USP related compound D was found unsatisfactory because a signal-to-noise ratio of only 20 was obtained for the impurity peak spiked at the 2.0% level, but this should be at least 20 times larger to obtain suitable sensitivity for the LOQ level (0.05%). In addition, non-polar organic solvents used in NP chromatography are toxic and should be avoided.

### 2.2. Method Scouting

The purpose of the work presented was to develop an HPLC method for efficient separation and determination of seven process-related impurities of celecoxib in the drug substance following AQbD principles [26]. The main issue that was encountered when testing the EP and USP methods was inadequate selectivity due to non-satisfactory separation between EP impurity A, EP impurity B, and celecoxib. Therefore, various stationary phases (BEH C18, HSS C18 SB, BEH Phenyl, HSS PFP, and CSH Fluoro-Phenyl from Waters; Force Biphenyl, Raptor Biphenyl, and Pinnacle DB BiPhenyl from Restek; Kinetex Biphenyl from Phenomenex; Zorbax SB Phenyl from Agilent; and Fortis Diphenyl from Fortis Technologies, Neston, UK) were investigated using acidic and neutral mobile phase pH in combination with acetonitrile or methanol in order to improve the selectivity. The p*K_a_* curves for EP impurity A, EP impurity B, and celecoxib molecules were very similar because the three molecules are positional isomers. The molecules are non-ionized in the pH range from 0.6 to 9.6 and have a p*K_a_* value around 10.6, and therefore pH should not have much effect on their separation.

Whereas EP impurity B was efficiently separated from celecoxib on several columns tested, obtaining a resolution of more than 2.0, the separation between EP impurity A and celecoxib was achieved only on the Zorbax SB Phenyl and Fortis Diphenyl columns, using an isocratic elution, with methanol as an organic modifier and a very high column temperature (≥60 °C). It was obvious that the additional π–π interactions between the stationary phase and the two analytes contributed to longer retention, and thus more efficient separation. Indeed, no effect of mobile phase pH was observed. However, the separation between EP impurity A and celecoxib on Zorbax SB Phenyl was still poor (resolution ≈ 0.7–1.0). Although the separation was better on Fortis Diphenyl (resolution ≈ 1.3–1.9), the tailing factor of the main peak was relatively high (tailing factor ≈ 2.9–3.4). Moreover, the column manufacturer does not recommend working at column temperatures above 60 °C, because this may decrease column efficiency, which is reflected in peak broadening and impairing the required resolutions.

Because Rao et al. reported a method in which positional isomers of celecoxib were successfully separated on a chiral column [16], selectivity was investigated using a Daicel Chiralpak AD-3R 150 × 4.6 mm column in RP mode. With a mobile phase consisting of acetonitrile/water in a 45/55% (*v*/*v*) ratio, separation between EP impurity A and celecoxib was achieved, but the USP related compound C eluted under the main peak.

**Figure 5:**
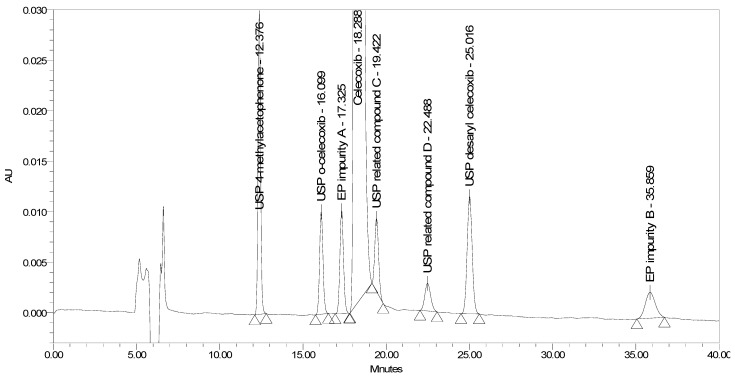
A chromatogram of celecoxib at a concentration of 0.5 mg/mL spiked with seven process-related impurities at the 0.5% level obtained using Chiralpak IA-3 with initial chromatographic conditions.

Instead of using a chiral stationary phase with traditionally coated silica support, a new generation of chiral columns was applied, in which a polysaccharide chiral selector with amylose tris (3,5-dimethylphenylcarbamate) derivative was immobilized on a silica support. The representative column was Chiralpak IA-3, 250 × 4.6 mm from Daicel. Using a mobile phase of acetonitrile/water in a 50/50% (*v*/*v*) ratio at a flow rate of 0.5 mL/min and column temperature of 35 °C, no co-elution of any impurity with the main peak was observed. However, the separation around the main peak, especially resolution between celecoxib and USP related compound C, which eluted on the tailing of celecoxib, was still non-satisfactory and needed improvement. A corresponding chromatogram is presented in Figure 5. Instead of acetonitrile, a combination of acetonitrile and methanol was also used as an organic modifier of the mobile phase. Introducing methanol to the mobile phase caused peak broadening and did not contribute to better resolution between critical peak pairs.

### 2.3. Analytical Target Profile, Critical Method Parameters, and Attributes

The first step in the AQbD approach, termed the analytical target profile (ATP), is the clarification of the purpose of the analytical method, including the definition of the measurement criteria that the method needs to be capable of fulfilling. A key element of the AQbD methodology is the proper selection of critical method attributes (CMAs). CMAs are the criteria that reflect the method performance defined in the ATP. The goal was to develop a method able to effectively separate all eight analytes in the shortest possible run time. On the basis of preliminary experiments performed during method scouting, the selected CMAs were resolution between EP impurity A and celecoxib (R1), resolution between celecoxib and USP related compound C (R2), resolution between USP related compound C and USP related compound D (R3), and retention time of EP impurity B (RT) as the last eluting impurity peak.

CMAs are influenced by variations of different factors called critical method parameters (CMPs). CMPs have to be properly set to fulfill the CMAs. For the method being developed, the ratio of acetonitrile in the mobile phase was the first CMP because it influences the selectivity. Column temperature was the second CMP, because it may also influence peak shape. The third CMP was flow rate, in order to minimize the analysis run time. The experiments from method scouting provided enough data to set the intervals for factor ranges and to set the detection wavelength and volume of injection, because they were not recognized as a CMP. The final factor ranges that needed to be investigated were 42–50% (*v*/*v*) of acetonitrile in the mobile phase, 30–40 °C for column temperature, and 0.5–1.0 mL/min for flow rate.

### 2.4. Method Optimization: Design of Experiments

The goal of method optimization was to achieve efficient separation of all eight analytes in the shortest possible run time. Design of experiments (DoE) [26,43,44] is a systematic approach to obtain a maximum amount of data with a minimum number of experiments performed, and it represents an extensive part of AQbD. Response-surface designs are the most appropriate for method optimization, because they enable to study each factor at three levels at least. Using DoE provides data for modeling. The aim of modeling is to determine the relationship between factors and responses, including factor interactions. The relation is described by a quadratic polynomial:

*y* = *b*_0_ + *b*_1_*x*_1_ + *b*_2_*x*_2_ + *b*_11_*x*_1_^2^ + *b*_22_*x*_2_^2^ + *b*_12_*x*_1_*x*_2_ + residual (1)

where *y* represents the measured response, *x*_1_ and *x*_2_ are the selected factors, *b*_0_ is the intercept, *b*_1_ and *b*_2_ are the coefficients for the linear (first-order) term, *b*_12_ is the coefficient for the interaction term, and *b*_11_ and *b*_22_ are the coefficients for the quadratic (second-order) term. Quadratic terms enable to model non-linear relationships, that is, to model curvature.

The DoE optimization of a method for determination of process-related impurities of celecoxib was performed by applying a central composite face response-surface design. The experimental design was created using MODDE Pro 11.0 software (Umetrics, Umeå, Sweden). For the investigation of 3 factors, 14 experiments were carried out, with the addition of 3 experiments in the center point to determine the experimental error. For each experiment, the four selected responses (CMAs) were measured. The experimental design with the measured responses is presented in Table 1.

The collected experimental data were processed using MODDE software. A multiple linear regression (MLR) method was applied to fit the mathematical model on the experimental data for each observed response, and non-significant factor terms were excluded from the model. The models created were statistically validated using analysis of variance (ANOVA) and were proven statistically significant (*p*-value < 0.05). High values of the coefficient of determination (*R*^2^) and adjusted coefficient of determination (adjusted *R*^2^) showed that the models created fit the experimental data adequately, and high values of the predicted coefficient of determination (*Q*^2^) indicate good prediction ability. In addition, excellent reproducibility of models for all responses was confirmed through replicated center point experiments, where the calculated pure error was negligible. The statistical significance and coefficients of determination of mathematical models for each response are presented in Table 2.

**Figure 6:**
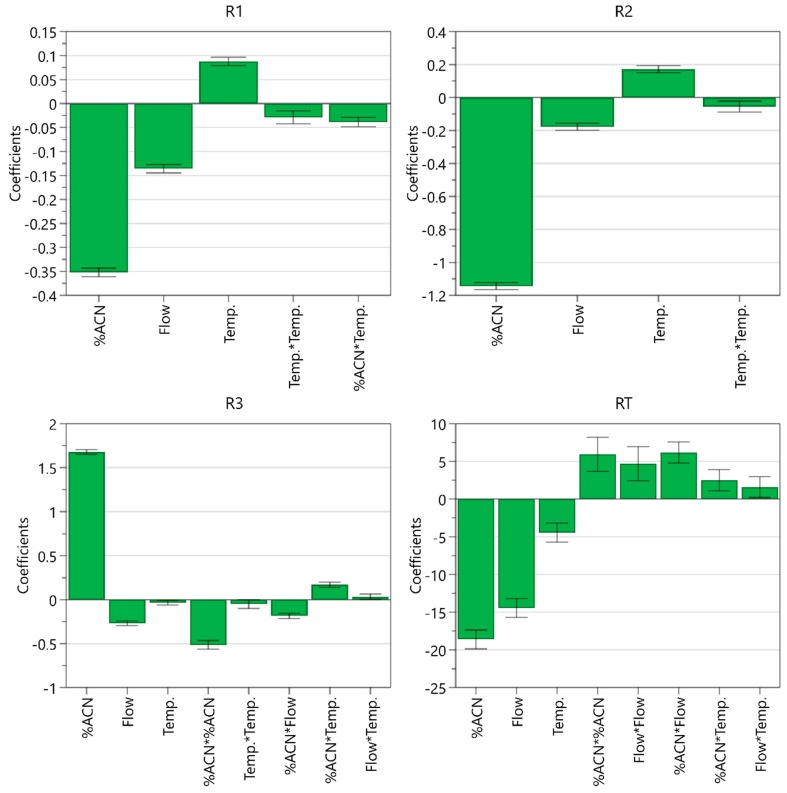
Coefficient plot for models created displaying their coefficients and confidence intervals.

From the coefficient plot (Figure 6), it can be seen that the ratio of acetonitrile in the mobile phase has the most significant effect on all CMAs. A higher percentage of acetonitrile decreases resolution between EP impurity A and celecoxib and resolution between celecoxib and USP related compound C, whereas it improves resolution between USP related compound C and USP related compound D and significantly shortens the retention time of EP impurity B. Flow rate is the second most influential CMP. Higher flow rate impairs all three resolutions but leads to shorter retention time of EP impurity B. On the other hand, higher column temperature improves the separation around the main peak. A significant negative acetonitrile ratio square term effect was also observed for resolution between USP related compound C and USP related compound D, whereas the effect of the square term was positive for the retention time of EP impurity B. Moreover, it was found that the interaction between the ratio of acetonitrile and flow rate influences resolution between USP related compound C and USP related compound D and retention time of EP impurity B, whereas the interaction between the ratio of acetonitrile and column temperature influences resolution between EP impurity A and celecoxib and resolution between USP related compound C and USP related compound D.

Because the goal of method optimization is to find the optimal combination of method parameters that yields the best method performance, the thresholds of acceptable values for CMAs have to be set. Based on preliminary experiments during method scouting and visual evaluation of obtained chromatograms, the CMA requirements for resolution were set to NLT 2.3 for resolution between EP impurity A and celecoxib (R1), NLT 3.2 for resolution between celecoxib and USP related compound C (R2) and NLT 2.0 for resolution between USP related compound C and USP related compound D (R3). In order to achieve reasonable run time, retention time for the last eluting impurity (EP impurity B) was limited to NMT 45 min (RT).

**Figure 7:**
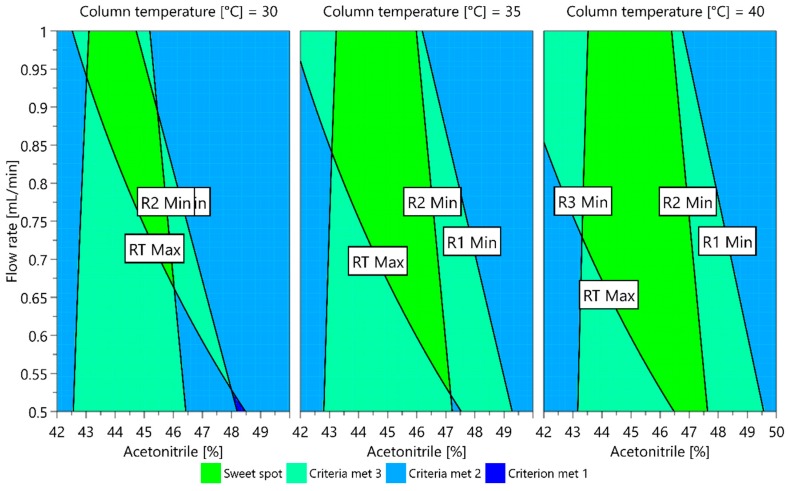
Sweet spot plot for a central composite design multiple linear regression (MLR) fitted model. The green area is a sweet spot where all CMA criteria are met.

The dependence of each CMA measured on the CMPs investigated can also be presented in graphic form as a contour plot. When contour plots are overlapped, a sweet spot plot is obtained, representing the areas where all CMA criteria are met and where one or more CMA requirements are not met. For the models obtained, a sweet spot plot was created using the MODDE integrated sweet spot analysis tool. The sweet spot plot is presented in Figure 7. The green area represents a sweet spot, a part where all CMA criteria are met, and the light green area is where one criterion is not met. From the plot presented, it can be seen that a higher column temperature offers a larger area where the changes in CMPs do not cause variation in CMAs outside their acceptable values. For example, using a column temperature of 40 °C, the ratio of acetonitrile in the mobile phase can vary between 43.5% and 46.5% if the flow rate is maintained between 0.7 mL/min and 0.9 mL/min to still fulfill the CMA requirements.

**Figure 8:**
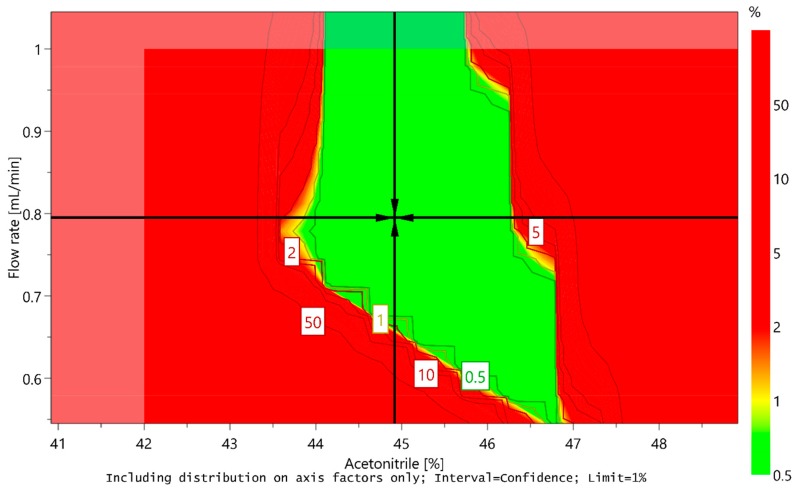
Design space for a central composite design MLR fitted model by plotting flow rate versus acetonitrile content at a column temperature of 40 °C. Predicted optimal conditions of critical method parameters (CMPs) are marked with a cross.

However, the sweet spot alone cannot ensure that the method will provide quality results with adequate probability, because it does not take into account possible variations deriving from the uncertainty of model-parameter estimates. Using a Monte Carlo simulation method, the risk for obtaining undesired results for each point in the sweet spot was calculated. Based on the calculations, a design space or method operable design region (MODR) was established, representing a robust region where there is a 99% certainty that all CMA criteria would be met. Moreover, optimal conditions of CMPs were predicted taking into account the predefined CMA criteria and their target values. The predicted optimal conditions proposed by MODDE are the ratio of acetonitrile in the mobile phase 44.918% (45%), flow rate 0.795 mL/min (0.8 mL/min), and column temperature 39.998 °C (40 °C). The design space is presented in Figure 8, and the predicted optimal conditions of CMPs are marked with a cross.

The optimal conditions of CMPs were experimentally verified and the responses measured were compared to the statistically predicted ones in MODDE (Table 3). The observed responses match the predicted values, and the relative difference is less than 1.0%. The final chromatographic conditions for the HPLC method developed are as follows:

- Chromatographic column: Chiralpak IA-3, 250 × 4.6 mm
- Column temperature: 40 °C
- Mobile phase: acetonitrile/water = 45/55% (*v*/*v*)
- Flow rate: 0.8 mL/min
- Detection: spectrophotometer at 250 nm
- Injection volume: 10 μL

**Figure 9:**
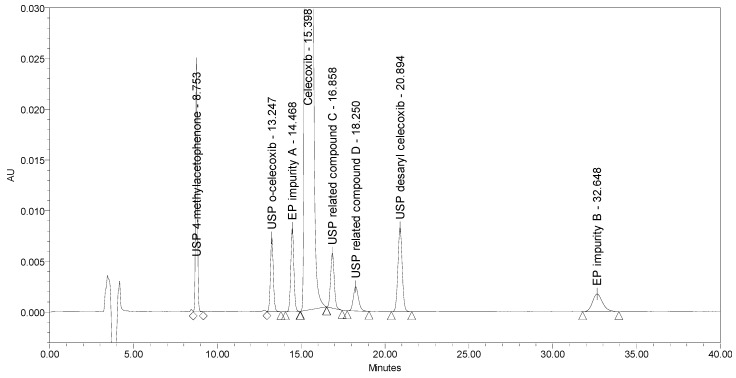
A chromatogram of celecoxib at a concentration of 0.5 mg/mL spiked with seven process-related impurities at the 0.5% level obtained using optimized chromatographic conditions.

The chromatogram obtained with these conditions is presented in Figure 9.

Even though robustness of the method is ensured in the design space, verification runs were performed according to a full factorial (2-level) screening design in order to experimentally verify whether small deliberate changes of CMPs will influence the quality of the established model. Factors were varied as follows: ±1.5% for acetonitrile content in the mobile phase, ±0.05 mL/min for flow rate, and ±1.5 °C for column temperature. Because the Daicel manufacturer prescribed a maximum column temperature of 40 °C, the center point for column temperature was set to 38.5 °C. Eight verification runs, including three experiments in the center point, were performed. The experimental design with the measured responses is presented in Table 4.

From Table 4, it can be seen that the CMA criteria were confirmed in each experimental run: NLT 2.3 for R1, NLT 3.2 for R2, NLT 2.0 for R3, and NMT 45 min for RT. Taking into account the resolution around the main peak (R1, R2), the most critical run was experiment N4 with a higher acetonitrile content and higher flow rate, but lower column temperature, which is in accordance with the effects presented in the coefficient plot (Figure 4). On the other hand, lower acetonitrile content and lower flow rate caused higher retention time (RT), as obtained in experiment N1, which is also in accordance with the coefficient plot. As expected, the column temperature is the least critical CMP. Even though the column temperature of 40 °C was determined as optimal, the method can also run at 38.5 °C without impairing the selectivity. This might prolong the column lifetime.

### 2.5. Method Verification

In the final step of the work presented, the method developed for determination of process-related impurities of celecoxib was verified in terms of precision, accuracy, linearity, limit of detection (LOD), and limit of quantification (LOQ). Method verification was performed according to ICH Q2(R1) guideline [45].

#### 2.5.1. Precision

The precision of the method was checked by injecting six replicates of sample solution of the drug substance. Because the celecoxib sample did not contain any impurity above the reporting limit, EP impurity A, the only specified impurity in EP monograph, was spiked to the sample at the 0.40% level (specification limit according to EP monograph). The results obtained for the six replicates yielded an intra-day RSD of impurity content being 1.01%. The result indicates suitable precision of the method.

#### 2.5.2. LOD and LOQ

LOD was determined by one injection of 0.1 µg/mL (0.02%) solution of celecoxib. The signal-to-noise ratio obtained was 9.5. LOQ was determined by six replicate injections of 0.25 µg/mL (0.05%) solution of celecoxib. The average signal-to-noise ratio was 24.2 with an RSD of celecoxib peak area being 0.50%. The determined LOD and LOQ were 0.02% and 0.05%.

#### 2.5.3. Linearity

The linearity of the method was verified in one replicate from LOD to 120% of specification limit of the highest specified impurity (EP impurity A), that is 0.48%. The stock standard solution was prepared with a concentration of approximately 0.25 mg/mL and was further diluted to obtain seven solutions of celecoxib with concentrations in the range 0.1–2.5 µg/mL (0.02–0.50%). A value of >0.9999 for the correlation coefficient demonstrated that the method is linear in the concentration range tested.

#### 2.5.4. Accuracy

The accuracy of the method was checked by injecting three sample solutions spiked with stock solution of each impurity investigated at three different concentration levels (LOQ, specification limit, and 120% of specification limit) in one replicate. The specification limit for EP impurity A was 0.40%, whereas the specification limit for all other impurities was 0.15%. The recoveries are presented in Table 5. The method was proven to be accurate.

#### 2.5.5. Response Factors

The response factors for each impurity were investigated in two replicates. Two stock solutions of each impurity with a concentration of approximately 0.1 mg/mL were prepared and further diluted to obtain solutions with a concentration of 2.5 µg/mL. The response factors were calculated from the areas of each impurity peak and the area of celecoxib peak obtained from 2.5 µg/mL standard solution, taking into account suitable potencies of the substances investigated. The response factors determined were 0.93 for EP impurity A, 0.76 for EP impurity B, 0.87 for USP related compound C, 0.45 for USP related compound D, 0.89 for USP o-celecoxib, 1.54 for USP desaryl celecoxib, and 2.00 for USP 4-methylacetophenone.

## 3. Materials and Methods

### 3.1. Reagents and Standards

Acetonitrile HPLC gradient grade (J.T.Baker, Radnor, PA, US), methanol (ultra) gradient HPLC grade (J.T.Baker, Radnor, PA, US), and purified water were used to prepare mobile phases and solvents.

An in-house celecoxib working standard (Lek, Ljubljana, Slovenia) was used. EP impurity A and EP impurity B standards were obtained from EDQM (Strasbourg, France). USP related compound C standard was obtained from USP (Rockville, MD, US). USP related compound D, USP o-celecoxib, and USP desaryl celecoxib standards were obtained from TLC Pharmaceutical Standards Ltd. (Ontario, Canada). USP 4-methylacetophenone chemical with a certificate was obtained from Merck (Darmstadt, Germany). Celecoxib drug substance was obtained from Cipla (Mumbai, India).

### 3.2. Diluent, Mobile Phase, Column, and Chromatographic Conditions

Diluent for the method developed consisted of methanol and purified water in a ratio of 75/25% (*v*/*v*). The mobile phase was composed of acetonitrile and purified water in a ratio of 45/55% (*v*/*v*). Separation was performed on Chiralpak IA-3 250 × 4.6 mm, 3 µm particle size column (Daicel, Tokyo, Japan). Isocratic elution at a flow rate of 0.8 mL/min was employed. The column temperature was maintained at 40 °C. Analytes were detected at 250 nm. The injection volume was 10 µL. For DoE purposes, the ratio of acetonitrile in the mobile phase, flow rate, and column temperature were varied as presented in Table 1 and Table 4.

### 3.3. HPLC System and Software

The experiments were performed on a Waters Alliance 2695 HPLC system (Waters Corporation, Milford, MA, US) consisting of a quaternary HPLC pump, auto sampler, and column thermostat, using a Waters 2489 UV/Visible detector. Instrument control and data acquisition were performed using Waters’ Empower 3 software (Waters Corporation, Milford, MA, USA). Creation of the experimental design and statistical analysis of results were performed using MODDE Pro 11.0 software (Umetrics, Umeå, Sweden). The p*K_a_* curves for the eight analytes investigated were calculated using MarvinSketch 17.28.0 software (ChemAxon, Budapest, Hungary).

### 3.4. Preparation of Solutions

#### 3.4.1. Preparation of Standard and Sensitivity Solutions

Twenty-five milligrams of celecoxib working standard was accurately weighed into a 100 mL volumetric flask. The substance was dissolved and diluted to volume with the diluent. The working standard solution was prepared by diluting 1.0 mL of the stock standard solution to 100 mL with the diluent, containing celecoxib at 0.0025 mg/mL. Sensitivity solution was prepared by diluting 1.0 mL of working standard solution to 10 mL with the diluent, containing celecoxib at 0.00025 mg/mL (0.05%).

#### 3.4.2. Preparation of Sample Solution

Twenty-five milligrams of celecoxib drug substance was accurately weighed into a 50 mL volumetric flask. The substance was dissolved and diluted to volume with the diluent.

#### 3.4.3. Preparation of Impurity Solutions

Solutions of impurities were prepared separately. For EP impurity A, EP impurity B, USP related compound C, USP related compound D, USP o-celecoxib, and USP desaryl celecoxib, 5 mg of impurity was accurately weighed into a 50 mL volumetric flask. The impurity was dissolved and diluted to volume with the diluent. For USP 4-methylacetophenone, 50 mg of impurity was accurately weighed into a 100 mL volumetric flask. The impurity was dissolved and diluted to volume with the diluent.

#### 3.4.4. Preparation of Spiked Solution for Method Optimization

Twenty-five milligrams of celecoxib drug substance was accurately weighed into a 50 mL volumetric flask, and 1.25 mL of EP impurity A, EP impurity B, USP related compound C, USP related compound D, USP o-celecoxib, and USP desaryl celecoxib solution, and 0.25 mL of USP 4-methylacetophenone solution were added. The spiked substance was dissolved and diluted to volume with the diluent. The resulting spiked sample contained each impurity at 0.5%.

### 3.5. Method Verification

An independent set of experiments with the method developed was carried out for method verification. The parameters assessed were precision, accuracy, linearity, limit of detection (LOD), and limit of quantification (LOQ). In addition, the response factors for all seven process-related impurities were determined.

## 4. Conclusions

In the work presented, AQbD principles were successfully applied to the development of a new HPLC method for the determination of seven process-related impurities of celecoxib. The pharmacopeial methods were not able to provide an adequate separation of two positional isomers from the main peak of celecoxib. The new method eliminates these issues; it is capable of efficiently separating and determining all seven impurities listed by the EP monograph and by the proposed USP monograph in one run.

Because an adequate separation of such similar analytes as positional isomers is often challenging, a systematic method optimization was necessary. To our knowledge, the case presented is the first that applies AQbD principles to the development and optimization of an impurity-profiling method for celecoxib drug substance. This brings several advantages to the new method. The DoE-supported method optimization facilitated a reduction of experimental work in comparison with the OFAT approach. Moreover, by creating the mathematical models for each CMA, detailed information on significant effects of factors and their interactions on a particular response was attained, which would not be possible with OFAT.

Obtaining information about method robustness is still the bottleneck of HPLC method development. In fact, the area of robustness for the pharmacopeial methods tested is unknown, which is especially inconvenient for the EP method, where the system suitability criterion could not be met, implying non-robust method performance. Applying the AQbD approach to the development of the new method, these issues were overcome because the area of method robustness was defined and 99% ensured in the design space. The area of method robustness was experimentally confirmed.

The method developed was verified in terms of precision, sensitivity, accuracy, and linearity, and it was proven to be fit for its intended purpose. In addition, using the AQbD approach, a robust method was developed in spite of the specific separations involved.

## Figures and Tables

**Table 1:** Central composite face design and the results of observed responses (critical method attributes, CMAs).

Exp. No.	Exp.	*x*_1_	*x*_2_	*x*_3_	R1	R2	R3	RT
1	N14	46	0.75	40	2.53	3.50	3.46	31.38
2	N17	46	0.75	35	2.45	3.37	3.55	34.56
3	N1	42	0.5	30	2.80	4.46	1.63	94.93
4	N10	50	0.75	35	2.11	2.25	4.72	23.16
5	N11	46	0.5	35	2.58	3.52	3.82	51.62
6	N5	42	0.5	40	3.04	4.84	1.18	75.61
7	N6	50	0.5	40	2.24	2.47	5.19	31.88
8	N12	46	1.0	35	2.31	3.21	3.29	26.04
9	N2	50	0.5	30	2.16	2.20	5.03	37.80
10	N15	46	0.75	35	2.47	3.37	3.59	34.70
11	N3	42	1.0	30	2.51	4.07	1.43	47.96
12	N4	50	1.0	30	1.89	1.80	4.02	18.96
13	N16	46	0.75	35	2.45	3.37	3.53	34.50
14	N13	46	0.75	30	2.32	3.20	3.49	37.98
15	N8	50	1.0	40	1.99	2.18	4.39	16.05
16	N7	42	1.0	40	2.76	4.46	1.04	38.41
17	N9	42	0.75	35	2.80	4.52	1.30	57.03

Exp. no. = number of experiment; Exp. = name of experiment; *x*_1_ = acetonitrile content in the mobile phase (%); *x*_2_ = flow rate (mL/min); *x*_3_ = column temperature (°C); R1 = resolution between EP impurity A and celecoxib; R2 = resolution between celecoxib and USP related compound C; R3 = resolution between USP related compound C and USP related compound D; RT = retention time of EP impurity B (min).

**Table 2:** Statistical significance and coefficients of determination of mathematical models for each response.

Model (Response)	*p*-Value (Regression)	*R* ^2^	*R*^2^ Adjusted	*Q* ^2^	Reproducibility
R1	8.5259 × 10^−16^	0.999	0.998	0.997	0.9986
R2	2.1878 × 10^−18^	0.999	0.999	0.998	1.0000
R3	5.9027 × 10^−13^	1.000	0.999	0.997	0.9995
RT	5.8067 × 10^−9^	0.996	0.993	0.963	1.0000

**Table 3:** Statistical model prediction for CMAs at optimal conditions of CMPs and experimentally obtained results.

CMA (Response)	Predicted	Observed
R1	2.59317	2.58367
R2	3.76787	3.73814
R3	2.89245	2.91747
RT	32.3846	32.648

**Table 4:** Full factorial screening design and the results of observed responses (CMAs).

Exp. No.	Exp.	*x*_1_	*x*_2_	*x*_3_	R1	R2	R3	RT
1	N6	46.5	0.75	40	2.47	3.37	3.69	29.67
2	N5	43.5	0.75	40	2.76	4.21	2.13	41.41
3	N10	45	0.8	38.5	2.57	3.7	2.97	33.77
4	N1	43.5	0.75	37	2.71	4.1	2.23	44.19
5	N4	46.5	0.85	37	2.38	3.25	3.66	27.84
6	N7	43.5	0.85	40	2.69	4.12	2.06	36.63
7	N11	45	0.8	38.5	2.56	3.69	2.95	33.64
8	N3	43.5	0.85	37	2.65	4.03	2.17	39.11
9	N8	46.5	0.85	40	2.41	3.3	3.59	26.21
10	N2	46.5	0.75	37	2.44	3.31	3.76	31.47
11	N9	45	0.8	38.5	2.58	3.71	2.97	33.82

Exp. no. = number of experiment; Exp. = name of experiment; *x*_1_ = acetonitrile content in the mobile phase (%); *x*_2_ = flow rate (mL/min); *x*_3_ = column temperature (°C); R1 = resolution between EP impurity A and celecoxib; R2 = resolution between celecoxib and USP related compound C; R3 = resolution between USP related compound C and USP related compound D; RT = retention time of EP impurity B (min).

**Table 5:** Results of recoveries at three concentration levels of investigated impurities of celecoxib.

Impurity	Concentration Level (%)	Added Conc. (µg/mL)	Found Conc. (µg/mL)	Recovery (%)
EP impurity A	0.05	0.2548	0.2632	103.31
	0.40	2.0380	2.0309	99.65
	0.48	2.4456	2.4255	99.18
EP impurity B	0.05	0.2498	0.2378	95.23
	0.15	0.7493	0.7468	99.67
	0.18	0.8991	0.8850	98.43
USP related compound C	0.05	0.2551	0.2073	81.26
	0.15	0.7653	0.6920	90.42
	0.18	0.9184	0.8442	91.92
USP related compound D	0.05	0.2637	0.2484	94.21
	0.15	0.7911	0.7753	98.00
	0.18	0.9493	0.9546	100.56
USP o-celecoxib	0.05	0.2690	0.2699	100.32
	0.15	0.8071	0.8128	100.71
	0.18	0.9685	0.9794	101.12
USP desaryl celecoxib	0.05	0.2455	0.2449	99.79
	0.15	0.7364	0.7375	100.16
	0.18	0.8836	0.8785	99.42
USP 4-methylacetophenone	0.05	0.2356	0.2371	100.65
	0.15	0.7067	0.7066	99.98
	0.18	0.8481	0.8452	99.66
